# Evaluation of Three Block Anesthesia Methods for Pain Management During Mandibular Third Molar Extraction: A Meta-analysis

**DOI:** 10.1038/srep40987

**Published:** 2017-01-20

**Authors:** Fanyuan Yu, Yao Xiao, Hanghang Liu, Fanzi Wu, Feng Lou, Dian Chen, Mingru Bai, Dingming Huang, Chenglin Wang, Ling Ye

**Affiliations:** 1 State Key Laboratory of Oral Diseases, West China Hospital of Stomatology, Sichuan University, Chengdu, Sichuan, China; 2 Department of Endodontics, West China Stomatology Hospital, Sichuan University, Chengdu, Sichuan, China.

## Abstract

A patient’s pain during mandibular third molar extraction often creates problems for a dental surgeon and can also cause immense patient discomfort, such as decreased quality of life, serious complications, or even danger to the patients’ lives. Effective pain management is therefore of great importance. Conventional block anesthesia method often fails to control such pain completely during an operation. Therefore, two available alternatives, Gow-Gates (G-G) and Vazirani-Akinosi (V-A) methods, have been developed. However, the results of current studies regarding their effectiveness and safety are somewhat ambiguous. The use of G-G and V-A techniques is therefore restricted. This study did a comprehensive review of the relevant research and finally 7 RCTs were included. The results of this meta-analysis indicate that both G-G and V-A techniques have a lower risk of positive aspiration. G-G technique also evidenced a higher success rate than the conventional method. V-A was faster while the G-G technique in contrast had a slower onset time than the conventional technique. In terms of the measurement of analgesic success, however, the V-A method was statistically indistinguishable from conventional techniques. These findings will hopefully endow clinicians with the knowledge required to make appropriate choices for effective anesthesia during lower third molar extraction.

More than 100 years of technical progress have generated progress in techniques of oral local anesthesia. Many kinds of small and medium oromaxillofacial surgeries are carried out on the premise of full analgesia^1^,^2^,^3^,^4^. The most popular anesthetic procedure used during the extraction of impacted mandibular third molars is the inferior alveolar nerve block (IANB), here referred to as the “conventional technique”, also known as direct mandibular nerve block or the Halstead technique. There are, in addition, several other major alternative approaches. The two which will be evaluated here are the Gow-Gates (G-G) and Vazirani-Akinosi (V-A) techniques^5^,^6^.

In 1884, Halstead and Hall first applied neuroregional anesthesia to the mandible, by injecting into the area of the mandibular foramina. The availability of this revolutionary injection has enabled dentists to deliver invasive dental treatments in a way that minimizes patient pain^7^. With the use of IANB, the nerve is accessed via the contralateral premolars from the opposite side of the mouth^8^. Another technique, the G-G technique, was invented by George A. E. Gow-Gates in the 1970s. It is characterized as follows: the needle is directed at the level of the neck of the condyle, which is located just under the insertion point of the lateral pterygoid muscle^9^. This technique is used for more extensive anesthesia or in cases where the IANB has not been successful. Yet another V-A technique was invented by Sunder J. Vazirani in 1960 and later reintroduced in 1977 by Oyekunle J. Akinosi. It is a closed-mouth injection technique in which the syringe is parallel to the maxillary occlusal plane at the level of the maxillary mucogingival junction^10^. This is often used when the patient cannot open his mouth wide enough for the IANB.

Even with the rapid development of techniques and materials for local anesthesia, however, administration of a single solution unfortunately does not always produce satisfactory pain management during mandibular third molar extraction^11^,^12^,^13^. But anesthetic impact can often be enhanced via the use of alternative approach routes or subsequent injections. Although these 3 techniques mentioned above are all available for clinicians, their relative effects and differential safety levels have not yet been definitively determined. It is therefore often difficult to choose the most appropriate procedure. Previous reports have indicated both advantageous and disadvantageous results along several dimensions^9^. Some studies consider G-G and V-A methods to be important supplements for IANB during third molar removal when the use of only IANB has resulted in anesthetic failure^14^. At present multiple randomized clinical trials (RCTs) have yielded ambiguous or contradictory results concerning both the validity and the safety of the new methods. Some studies report no significant differences among these 3 anesthetic methods in terms of success rate, onset time, or positive aspiration rate. On the other hand there are other studies whose results indicate that G-G and V-A methods enjoy certain advantages over the traditional IANB, though the latter is often considered to be the default technique that should be selected^6^,^15^,^16^,^17^,^18^,^19^,^20^. These contradictory research results create hesitation with respect to G-G or V-A; in view of research ambiguity, many dentists are reluctant to adopt these two techniques. There is therefore an urgent need to evaluate the comparative anesthetic effectiveness and safety of these 3 techniques^7^.

This study reviews the results of research that had been carried out prior to December 2015 and uses meta-analysis to examine relative effectiveness concerning three outcome variables: anesthetic success rate, anesthetic onset time and the occurrence of bleeding on plunger extraction (here referred to as positive aspiration rate). With respect to the first two, enhanced anesthetic success rate and shorter onset time increase receptivity to a technique. And a decrease in the risk of positive aspiration not only enhances the safety but also the effectiveness of local anesthesia. These 3 outcome variables therefore are here used as the most important indicators for evaluating the comparative effectiveness of different anesthetic techniques. Research into these variables can hopefully serve as a useful empirical guide to practicing surgeons. Briefly, this study is aimed to evaluate the effect and safety between G-G/V-A and IANB for the pain management during mandibular third molar extraction.

## Results

### Search Results

A total of 232 studies surfaced during the search procedures mentioned above. After selection according to the inclusion and exclusion criteria, 7 RCTs were included for quantitative synthesis (meta-analysis)^21^,^22^,^23^,^24^,^25^,^26^,^27^. (flow diagram was shown in Supplemental data).

### Characteristics of included studies

7 randomized controlled trials (RCTs) with 3 split mouth designs and 4 parallel designs were included; data from these studies were extracted, pooled and then meta-analyzed. These publications date from 1896 to 2013. Among split trials, Allen L 1896 compared IANB and V-A with respect to their pain control success rate, their onset time and their positive aspiration rate^21^. Jiacai He 2000 treated the IANB as a control and utilized V-A as the experimental intervention; the study evaluated the success rate and the positive aspiration rate. (There were 40 patients in total, with an age range of 22–38 and a mean age of 29.5. The female/male ratio was 16/24)^22^. Jieping Yang 2013 defined G-G (rather than V-A) as the experimental treatment. (There were 32 patients in all, with a mean age of 11.8–38.2 and a female/male ratio of 18/14)^23^. This study estimated the same outcome variables as the above-mentioned Allen L 1896 study.

As for the following parallel design trials, Diandian Li^24^ included 420 patients, utilizing IANB as the control treatment whereas patients receiving V-A and G-G were treated separately as the experimental groups, utilizing success rate, onset time and positive aspiration rate as the outcome variables to be evaluated. (The age range was 18–38, with a mean age of 29.5 and a female/male ratio of 163/257). Fei Wang 2002 also adopted a parallel design and assessed the same outcome variables as Diandian Li 2009. In contrast, however, this study compared G-G with IANB including 30 patients for each group. (The age range was 18–35, with a mean age of 26, and a female/male ratio of 18/42)^25^. A study by Jizhong Lv 2009 measured all three outcomes mentioned above on a sample of 120 patients. In this study the outcome of the V-A treatment was compared to that of the IANB. (The age range was 18–38 with a mean age of 29.5 and a female/male ratio of 47/73)^26^. Similarly, Martinez-G 2003 studied these three outcomes on a sample of 120 patients that received either the V-A or the IANB (The age range was 18–38, with a mean age of 29.5 and a female/male of 47/73)^27^. In contrast, Mohammad RJ 2013, which met all inclusion criteria, adopted a split mouth design and defined patients receiving IANB as the control group and patients receiving G-G as the experimental treatment group^28^. But it did not present any of the outcomes analyzed in this study, so it unfortunately had to be excluded (Table 1).

### Assessment of the risk of bias

**Figure 1:**
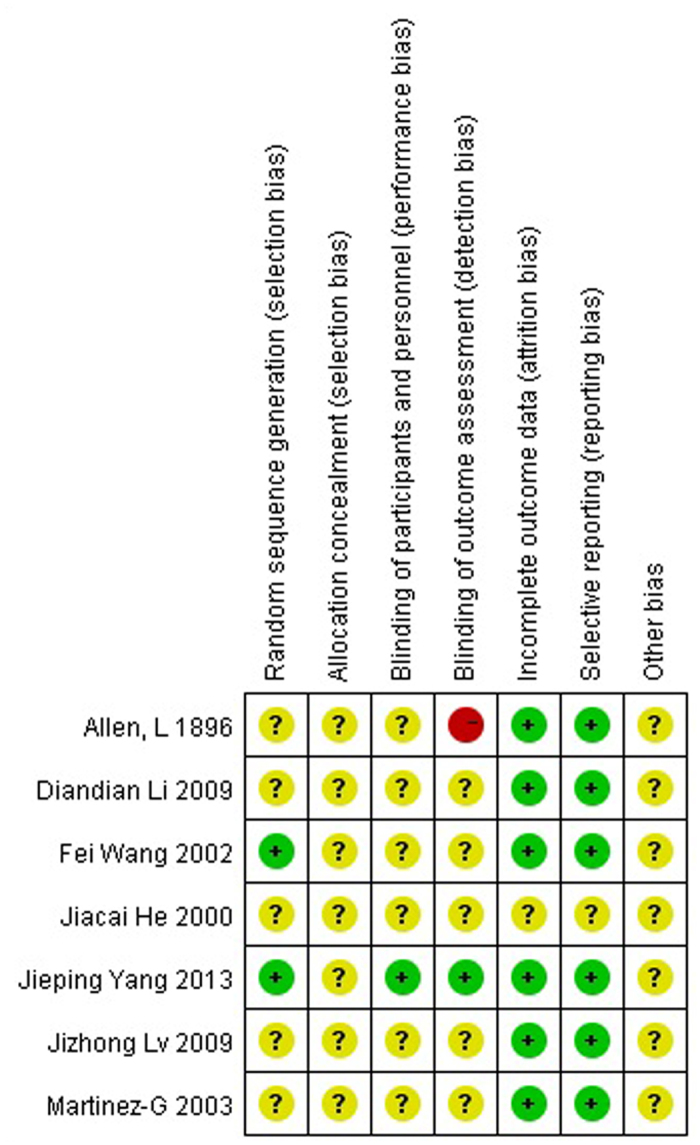
Risk of bias summary: review authors’ judgments about each risk of bias item for each included study.

Within the RCTs included for meta-analysis, only one of them showed a high risk of bias while the remainder exhibited a moderate risk of bias. Since the surgeon not only performed the operation but also assessed the outcomes in Allen, L 1896, a detection bias may have existed due to a violation of blinding of outcome assessment. Moreover, several domains were judged as unclear in this study because of the lack of information. (Figure 1) Besides, combination of split design and parallel design did not increase heterogeneity in every outcome. Contrarily, removal of split design increased the heterogeneity in the comparison of positive aspiration between G-G and IANB. Hence, we combined split and parallel designs in this study (S7–12).

## Efficacy of These Three Techniques

### Estimation of onset time

**Figure 2:**
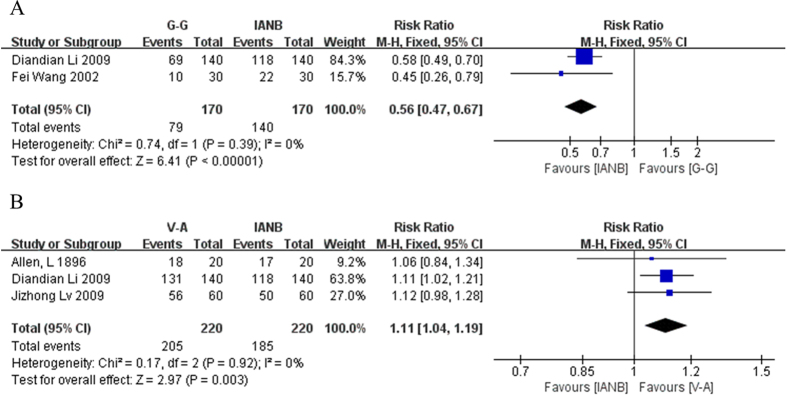
(**A**) Forest plot of comparison: G-G method was compared with IANB method, outcome: onset time. (**B**) Forest plot of comparison: V-A method was compared with IANB method, outcome: onset time.

With respect to onset time, two studies compared the results of the G-G and the IANB methods. Patients in each study were divided into two groups. In one group the onset time of analgesic effect was less than 5 minutes. In the other group it took longer. Diandian Li 2009 reported that 69 patients in the G-G group felt no pain within 5 minutes of injection, whereas the IANB had 118 patients who fell into this category. (Each group had 210 patients). Additionally Fei Wang 2002 reported that whereas only 10 patients in the G-G group achieved successful analgesia within 5 minutes, 22 in the IANB group did. (Each group had 30 members). The data from these two studies were pooled and analyzed statistically. The results indicated that the conventional IANB technique has a statistically significant faster analgesic onset time than the G-G method (RR = 0.56, 95% CI = 0.47–0.67, P < 0.00001). In addition, the study documented a low level of heterogeneity (χ^2^ = 0.74, P = 0.39, I^2^ = 0%). However, three other studies showed that the V-A treatment had a more rapid onset time than the IANB method. In these three studies (Allen,L 1896, Diandian Li 2009 and Jizhong Lv 2009) the numbers of patients whose onset time was shorter than 5 minute were respectively 18, 131 and 56, with the control group respectively contained 17, 118 and 50. The results of meta-analysis concluded that the V-A method has a more rapid onset time than the IANB method (RR = 1.11, 95% CI = 1.04–1.19, P = 0.003) with low heterogeneity (χ^2^ = 0.17, P = 0.92, I^2^ = 0%) (Fig. 2).

### Evaluation of success rate

6 studies reported on the pain-killing success of the anesthesia. In these studies, patients were divided into three categories according to their score on the following-mentioned Dobbs scale: mild pain, moderate pain with no need for supplemental anesthesia, severe pain requiring supplemental anesthesia to complete the procedure. We defined mild and moderate pain as a successful anesthetic outcome and severe pain as anesthetic failure. Diandian Li 2009 reported that 140 patients in the V-A and G-G group achieved successful anesthesia whereas the IANB had 136 patients with a successful outcome. In another study, Jiacai He 2000 reported that 38 patients in the V-A group, and 37 in the IANB group achieved successful anesthesia. Jizhong Lv 2009 reported that 60 patients in the V-A group and 58 in the IANB group did not need supplemental anesthesia. Martinez-G found that 23 patients in the V-A group and 25 in the IANB group achieved successful anesthesia. Fei Wang 2002 recorded 29 and 27 successfully anesthetized patients in the G-G and the IANB groups respectively. Jieping Yang 2013 found that 31 patients in the G-G group and 29 in the IANB group were successfully anesthetized.

**Figure 3:**
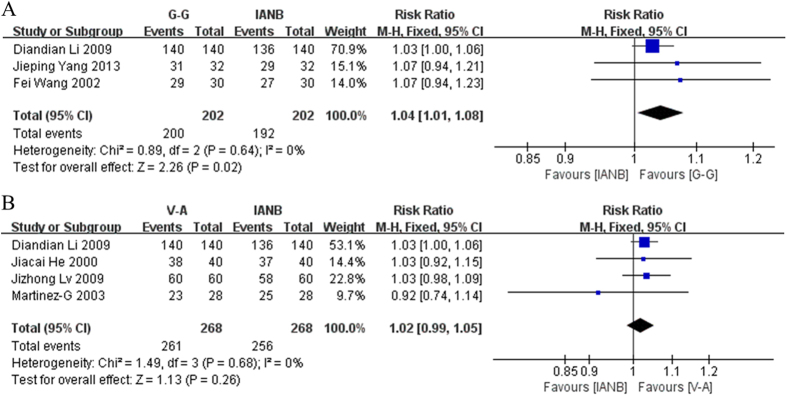
(**A**) Forest plot of comparison: G-G method was compared with IANB method, outcome: success rate. (**B**) Forest plot of comparison: V-A method was compared with IANB method, outcome: success rate.

Data from these studies were pooled and statistically analyzed. The G-G technique proved to have a statistically higher rate of successful anesthesia than the IANB method. (RR = 1.04, 95% CI = 1.01–1.08, P = 0.02.) In addition, this meta-analysis showed low heterogeneity (χ^2^ = 0.89, P = 0.64, I^2^ = 0%). On the other hand, there was no statistical significance between the V-A and IANB methods (Fig. 3).

## Safety of These Techniques

### Risks of positive aspiration

**Figure 4:**
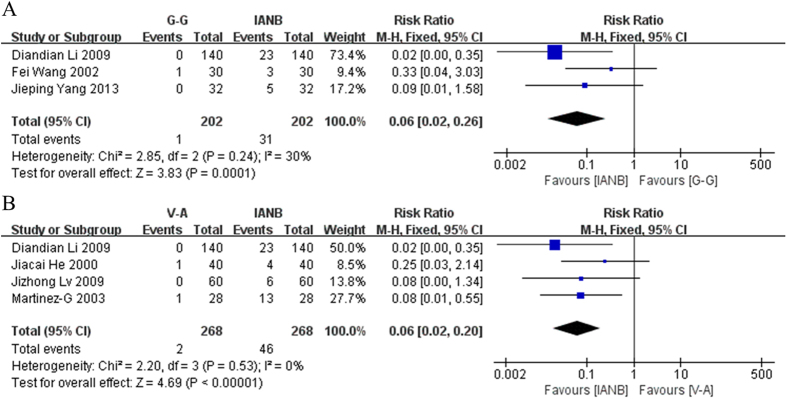
(**A**) Forest plot of comparison: G-G method was compared with IANB method, outcome: positive aspiration rate. (**B**) Forest plot of comparison: V-A method was compared with IANB method, outcome: positive aspiration rate.

6 trials reported on the positive aspiration rate. Patients were divided into two groups: those that experienced positive aspiration and those that did not. Diandian Li 2009 reported that whereas 0 patients receiving V-A or G-G anesthesia tested positive for aspiration, 23 patients receiving IANB did so. Additionally, Jiacai He 2000 found that only 1 patient receiving V-A, but 4 receiving IANB experienced positive aspiration. Jizhong Lv 2009 reported that 0 patients in V-A and 6 in IANB group experienced positive aspiration. Martinaz-G reported that 1 patient in the V-A group and 13 in the IANB group had positive aspiration. Fei Wang 2002 reported that 1 and 3 patients in the G-G and IANB groups respectively experienced positive positive aspiration. Jieping Yang 2013 found that 0 patients in the G-G group and 5 in the IANB group had positive aspiration. The data from these studies were pooled and analyzed statistically. The results showed that the G-G and V-A techniques have a statistically significant lower incidence of positive aspiration than the IANB method. (G-G: RR = 0.06, 95% CI = 0.02–0.26, P = 0.0001; V-A: RR = 0.06, 95% CI = 0.02–0.20, P < 0.00001). In addition, both of them yielded low heterogeneity (G-G: χ^2^ = 2.85, I^2^ = 30% ; V-A: χ^2^ = 2.20, I^2^ = 0%) (Fig. 4).

## Incidence of Other Complications

Fei Wang 2002 reported that 1 patient in the IANB and G-G groups respectively reported pain in the injection area, and 1 patient in the IANB group experienced swelling. Jizhong Lv 2009 reported that 1 patient in the IANB group experienced pain in the injection area and 2 patients manifested swelling. In contrast, no complications were found in the V-A group.

## Discussion

Successful local anesthesia is required for control of pain during dental procedures. Anesthesia should also occur with a low rate of additional complications, such as positive aspiration, swelling and so on. The inferior alveolar nerve block is the most commonly used block during oral surgery. However, this block has a comparatively high failure rate^29^, mainly because of inter-patient anatomic variations. Therefore, various other nerve blocks were utilized over time to improve the success rate of anesthesia. The G-G and V-A methods discussed in this paper have been the most important alternatives. They differ from the conventional IANB technique in terms of the location of the injection point. From the analysis of the RCTs included above, it can be concluded that G-G and V-A techniques enjoy several advantages over IANB techniques when utilized during mandibular third molar extraction. The results suggest that they may be a useful alternative to IANB with regard to an increase in anesthetic success and a decrease in clinically dangerous positive aspiration.

Some studies even suggest that these alternative techniques may decrease the incidence of certain complications such as swelling and pain in the injection area. With respect to these ancillary advantages of the alternative techniques, however, not enough research has been done to conclude via meta-analysis that G-G or V-A can de facto reduce such complications.

The meta-analysis has, however, uncovered one enigma: the analgesic onset time of both G-G and V-A is longer than of IANB. The reasons for this are not clear. But because they differ from the IANB technique principally in terms of the location of various injection points, one could hypothesize that a slower onset time might be a result of different anatomic factors associated with different injection areas. Among these factors could be differences in nerve distribution, in blood flow condition, or even in soft tissue conditions associated with distinct injection areas.

In IANB the anesthetic is injected in the pterygo-submandibular space, the posterior boundary of which is the parotid gland, exterior the ramus of the mandible, interior and inferior the medial pterygoid, superior the external pterygoid, and anterior the masseter. The G-G method consists somewhat of a type of “high” inferior alveolar nerve block anesthesia, whose anesthetic is injected principally at the mandibular condylar region. In the V-A approach, the anesthetic is injected into the upper part of the pterygo-mandibular space, so that the three branches of the mandibular nerve (the inferior alveolar, lingual and buccal nerves) are anesthetized.

It should also be pointed out that, due to various confounding variables, such as types and concentrations of anesthetic substances adopted in these seven RCTs, as well as differential levels of experience on the part of those administering the anesthesia, it is not possible to formulate absolute conclusions concerning their properties of onset time, success rate and positive aspiration rate. Four of the studies used lidocaine, one adopted articaine and two did not describe the drug type. ALLEN L 1896 conducted local anesthesia with administration of 8 ml 2% lidocaine in V-A while using 2.3 ml in the IANB group. Fei Wang 2002 utilized 3 ml 2% lidocaine in both the IANB and the G-G groups. Jieping Yang 2013 utilized 3–5 ml 2% lidocaine in both the IANB and the G-G groups. Jiacai He 2000 used 2 ml 2% lidocaine in both IANB and V-A. Martinez, G 2003 used 1.8 ml 4% articaine in both IANB and V-A. Jizhong Lv 2009 and Diandian Li 2009 did not provide specific details about anesthetic types and concentrations in each group. Apart from these variations among the studies, the issue of different concentrations of epinephrine also arises. Martinez, G 2003 ALLEN L 1896 and Jieping Yang 2013 adopted a 1:100,000 concentration of epinephrine. Jiacai He 2000 also added an unspecified amount epinephrine in the anesthesia. The remaining three RCTs had no information on this matter. It is true that previous research showed that the concentration of epinephrine does not influence the anesthetic success rate of IANB^30^. However, there is not enough evidences to indicate whether epinephrine concentration will affect the anesthetic effectiveness and safety of the G-G and V-A methods. Variations in these factors might lead to a higher index of heterogeneity, which could arguably weaken the results of this meta-analysis.

Despite the presence of these limitations, however, this meta-analysis has successfully compared intervention and control techniques under similar conditions. It seemed both logical and valid to pool together data from these studies. On the other hand, further studies should be undertaken that would take into consideration variables such as anesthetic and epinephrine types and concentrations to verify these findings.

## Conclusion

In order to confirm conclusions of this meta-analysis further large-scale RCTs with similar protocols and strict randomization and blinding designs need to be conducted. Overall, G-G and V-A techniques had a lower risk of positive aspiration than IANB. This difference can be interpreted as an important indicator of a higher level of safety. G-G also showed a higher success rate in terms of analgesic effect but, as pointed out before, manifested a longer onset time than IANB. This method can therefore be recommended for pain management during mandibular third molar extraction, but it may require that the surgeon wait longer before the operation. The V-A technique demonstrated a shorter onset time but there was no statistically significant difference between V-A and IANB in terms of a successful anesthetic effect. This paper concludes, therefore, by suggesting that V-A can be regarded as an adequate replacement for IANB in cases where the latter fails to achieve the desired pain control effect during lower third molar extraction.

## Methods

This meta-analysis was performed according to a previously developed protocol; it was carried out in accordance with Preferred Reporting Items for Systematic Reviews and Meta-Analyses (PRISMA) and the Cochrane Handbook for Systematic Reviews of Interventions^31^,^32^.

## Inclusion Criteria

### Types of studies

All RCTs, including split mouth RCTs and parallel RCTs, were considered for inclusion in this review. Quasi-RCTs, controlled clinical trials (CCTs), cohort studies, case reports and other studies that fall outside of the category RCT were excluded.

### Types of participants

In accordance with the Guidelines of the National Institute for Health and Clinical Excellence (NICE)^33^, participants were chosen from among those patients who had been referred for removal of the mandibular third molar and who did not suffer from any of the following conditions: local infection, severe periodontal diseases, acute pericoronitis, or related conditions in adjacent teeth. In addition, patients on preoperative medication with anti-inflammatory drugs, such as nonsteroidal anti-inflammatory drugs (NSAIDs), and/or analgesics were excluded from the research, as were those suffering from systematic diseases that potentially affect anesthetic effectiveness or safety. (Patients with hypertension are one such example).

### Types of interventions

Patients receiving the conventional treatment (namely IANB) were treated as the control group. They were compared with patients receiving either the Gow-Gates technique, the Vazirani-Akinosi technique, or both. Special modifications introduced in the application of these treatments were not taken into account.

### Types of outcome measures

#### Primary outcomes

This analysis was designed to measure the differential pain control effectiveness and the differential safety of IANB, G-G and V-A anesthetic methods during mandibular third molar extraction. Anesthetic onset time, anesthetic success rate, and positive aspiration rate are treated as the major outcome variables.

#### Incidence of other complications

Pain at the injection area, post-operative swelling, syncope, toxication, post-operative pain or other complications, if mentioned in the RCTs, will also be discussed but not utilized in the meta-analysis.

## Exclusion Criteria

Published clinical trials were excluded if they did not meet the above criteria.

## Search Methods

The search was restricted to articles written in English and Chinese. A literature search was carried out within the Cochrane Central Register of Controlled Trials (CENTRAL; 2015), MEDLINE (via OVID, 1948 to December 2015), Embase (1984 to December 2015), the China National Knowledge Infrastructure (CNKI; 1979 to December 2015), and the China Biology Medicine disc (CBM; 1978 to December 2015). The online databases of the British Journal of Oral and Maxillofacial Surgery, the Journal of Oral and Maxillofacial Surgery, the International Journal of Oral and Maxillofacial Surgery and the Journal of Dental Research were also searched. Manual searches were also carried out in relevant Chinese journals. Reference lists of relevant articles were checked. In order to find ongoing clinical trials, the World Health Organization International Clinical Trials Registry Platform was searched. The MeSH heading words and free text words were combined. They included “third molar”, “wisdom tooth”, “impacted tooth”, “unerupt tooth”, “mandibular molar”, “tooth extraction”, “remove tooth”, “pain”. We combined these words with synonyms for “inferior alveolar nerve block”, “IANB”, “conventional technique”, “direct mandibular nerve block”, “Halstead technique”, “Gow-Gates technique” or “Vazirani-Akinosi”. Search strategies were finally combined with the Cochrane Highly Sensitive Search Strategy to identify randomized trials. Reference lists of the retrieved articles were also checked.

## Study Inclusion

Independently, three reviewers (YFY, LHH and XY) screened and evaluated the titles and abstracts of all potential articles according to the pre-established selection criteria. Then full-texts were further assessed for all studies that possibly met the inclusion criteria or for cases in which it was difficult to make a final decision because of insufficient information. When disagreements came up, they were resolved by consensus, and an alternative investigator (YL or WCL) acted as an arbiter when no consensus was reached.

## Assessment of Risk Of Bias

According to the guidelines from Cochrane, we assessed selection bias, performance bias, detection bias, attrition bias and reporting bias. The Cochrane “risk of bias” instrument was used to assess the risk of bias. This evaluation was performed by 3 independent reviewers (YFY, LHH and XY). Disagreements between estimators were resolved by discussion until consensus was reached. The risk of bias was classified into three categories:

(a) Low risk of bias if all domains were marked as “low risk”;

(b) Moderate risk of bias if no domain was marked as “high risk” but at least one was coded as “unclear risk”;

(c) High risk of bias if more than one domain was marked as “high risk”.

## Data Extraction and Data Pooling

The following data were extracted: demographic data, method of randomization, randomization concealment and blinding, outcomes (success rate, onset time, positive aspiration rate). Two estimators independently extracted data from the included studies (YFY and LHH) using a custom-designed form.

Besides, this analysis was designed to measure the differential pain control effectiveness and the differential safety of IANB, G-G and V-A anesthetic methods during mandibular third molar extraction. Anesthetic onset time, anesthetic success rate, and positive aspiration rate are treated as the major outcome variables. To measure the onset time of anesthesia all patients were divided into two groups: those who experienced analgesia within 5 minutes, and those who took longer. For a study to be included, patients’ pain condition had to be explicitly described, either in terms of the surgeon’s evaluation of the patient’s pain state or of the patient’s self-evaluation. Success rate is defined as successful pain management during the entire operation. The following conditions will be recognized as indicators of success: either sufficient analgesic duration time to complete the extraction surgery, or achievement of an A or B grade on the Dobbs classification of anesthetic depth. Grade A indicates complete analgesia during the entire surgery; grade B indicates a slight, tolerable amount of pain that requires no further anesthesia. Grade C indicates a high level of pain that required additional anesthesia^34^. In addition, if alternative scales, such as the Visual Analogue Scale (VAS) or similar classification tools, were adopted in a trial, they were converted into a comparable Dobbs’ grade based on the author’s description. The positive aspiration rate is one of the most important factors that influence oral local anesthesia. Hence, the absence of positive aspiration will be regarded as the prime indicator of safety.”

## Assessment of Heterogeneity

Overall, clinical heterogeneity was assessed qualitatively. Patients, design, setting, and intervention characteristics were taken into consideration. Methodological heterogeneity was evaluated via the Risk of Biastool^35^. Efforts were made, where possible, to estimate reporting biases according to the recommendations from the Cochrane Collaboration tool^36^.

## Statistical Analysis

Statistical analyses were carried out utilizing Review Manager 5.1. Heterogeneity was assessed via the I^2^ statistic (a test for heterogeneity) on the level of α = 0.10. If there was considerable or substantial heterogeneity (I^2^ > 50%), a random-effects model was adopted; otherwise a fixed-effects model was used. The results of treatment effect were presented as risk ratio (RR) utilizing 95% confidence intervals (CIs). Statistical significance was calculated at α = 0.05 (2-tailed z tests). Odds Ratio (OR) and RR can both measure outcomes in our study without significant mistakes neither. But RR will make the measurements better so we adopted RR in this study (S1–6).

## Additional Information

**How to cite this article:** Fanyuan, Y. *et al*. Evaluation of Three Block Anesthesia Methods for Pain Management During Mandibular Third Molar Extraction: A Meta-analysis. *Sci. Rep.* **7**, 40987; doi: 10.1038/srep40987 (2017).

**Publisher's note:** Springer Nature remains neutral with regard to jurisdictional claims in published maps and institutional affiliations.

## Supplementary Material

Supplemental Data

## Figures and Tables

**Table 1:** Characteristics of included studies.

Study Id	Patient			Intervention		Outcomes	Study tpye (study design)
	Number	Age(Year)	Gender (F/M)	Technique	I/C		
Allen, L 1896	20	—	—	C:IANB, I:V-A	20/20	success rate, onset time, positive aspiration	RCT(split mouth)
Diandian Li 2009	420	M = 29.5, Range:18–38	163/257	C:IANB, I:V-A, G-G	140/140/140	success rate, onset time, positive aspiration	RCT(parallel)
Fei Wang 2002	60	M = 26.0, Range:18–35	18/42	C:IANB, I:G-G	30/30	success rate, onset time, positive aspiration	RCT(parallel)
Jiacai He 2000	40	M = 29.5, Range:22–38	16/24	C:IANB, I:V-A	40/40	success rate, positive aspiration	RCT(split mouth)
Jieping Yang 2013	32	M = 25.0, Range:11.8- ± 38.2	18/14	C:IANB, I:G-G	32/32	success rate, onset time, positive aspiration	RCT(split mouth)
Jizhong Lv 2009	120	M = 29.5, Range:18–38	47/73	C:IANB, I:V-A	60/60	success rate, onset time, positive aspiration	RCT(parallel)
Martinez, G 2003	56	M = 23.3, Range:14–38	34/22	C:IANB, I:V-A	28/28	success rate, onset time, positive aspiration	RCT(parallel)

—: no information; M: mean; F/M: Female number versus male number; C: control group, I: intervention group; I/C: patients number in intervention group versus the number of control group.
