# Supplementary material for: Evaluation of Three Block Anesthesia Methods for Pain Management During Mandibular Third Molar Extraction: A Meta-analysis
Source: Sci Rep. 2017 Jan 20;7:40987. doi: 10.1038/srep40987 (PMC5247732; doi:10.1038/srep40987)

Supplemental data for response

Evaluation of Three Block Anesthesia Methods for Pain Management During Mandibular Third Molar Extraction: A Meta-analysis

Yu Fanyuan[1*], Xiao Yao[1*], Liu Hanghang[1], Wu Fanzi[1], Lou Feng[1], Chen Dian[1], Bai Mingru[1], Huang Dingming[12], Wang Chenglin[12#], Ye Ling[12#]

1. State Key Laboratory of Oral Diseases, West China Hospital of Stomatology, Sichuan University, Chengdu, Sichuan, China
2. Department of Endodontics, WestChina Stomatology Hospital, Sichuan University

* Yu Fanyuan and Xiao Yao contributed equally in this work.
# Joint Corresponding authors: Wang Chenglin and Ye Ling.

Wang Chenglin:
State Key Laboratory of Oral Diseases, Sichuan University, Chengdu, Sichuan, China.
Department of Endodontics, WestChina Stomatology Hospital, Sichuan University, No.14, Section 3, South Renmin Road, Chengdu, China, 610041.
Email: wxonet@163.com

Ye Ling: DDS, PhD, Department of Endodontics, West China Stomatology Hospital, Sichuan University, No.14, Section 3, South Renmin Road, Chengdu, China, 610041.
E-mail: yeling@scu.edu.cn

S1

Onset time(G-G vs IANB)

	G-G		IANB			Odds Ratio	Odds Ratio
**Study or Subgroup**	**Events**	**Total**	**Events**	**Total**	**Weight**	**M-H, Fixed, 95% CI**	**M-H, Fixed, 95% CI**
Diandian Li 2009	69	140	118	140	80.3%	0.18 [0.10, 0.32]	
Fei Wang 2002	10	30	22	30	19.7%	0.18 [0.06, 0.55]	
**Total (95% CI)**		**170**		**170**	**100.0%**	**0.18 [0.11, 0.30]**	
Total events	79		140				

Heterogeneity: Chi² = 0.00, df = 1 (P = 1.00); I² = 0%
Test for overall effect: Z = 6.66 (P < 0.00001)

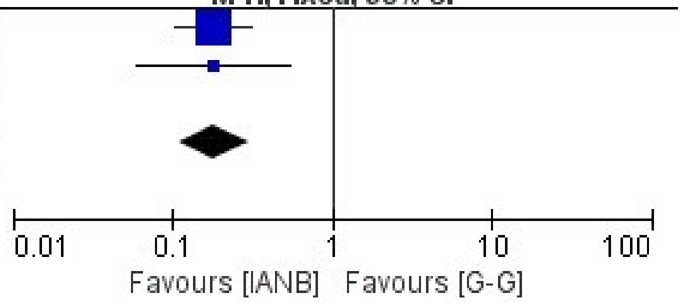

	G-G		IANB			Risk Ratio	Risk Ratio
**Study or Subgroup**	**Events**	**Total**	**Events**	**Total**	**Weight**	**M-H, Fixed, 95% CI**	**M-H, Fixed, 95% CI**
Diandian Li 2009	69	140	118	140	84.3%	0.58 [0.49, 0.70]	
Fei Wang 2002	10	30	22	30	15.7%	0.45 [0.26, 0.79]	
**Total (95% CI)**		**170**		**170**	**100.0%**	**0.56 [0.47, 0.67]**	
Total events	79		140				

Heterogeneity: Chi² = 0.74, df = 1 (P = 0.39); I² = 0%
Test for overall effect: Z = 6.41 (P < 0.00001)

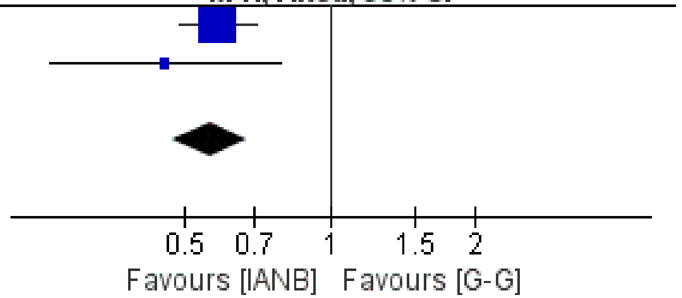

S2

Onset time(V-A vs IANB)

Study or Subgroup	V-A Events	V-A Total	IANB Events	IANB Total	Weight	Odds Ratio M-H, Fixed, 95% CI
Allen, L 1896	18	20	17	20	13.5%	1.59 [0.24, 10.70]
Diandian Li 2009	131	140	118	140	60.1%	2.71 [1.20, 6.13]
Jizhong Lv 2009	56	60	50	60	26.4%	2.80 [0.83, 9.49]
**Total (95% CI)**		**220**		**220**	**100.0%**	**2.58 [1.37, 4.89]**
Total events	205		185			

Heterogeneity: Chi² = 0.28, df = 2 (P = 0.87); I² = 0%
Test for overall effect: Z = 2.92 (P = 0.003)

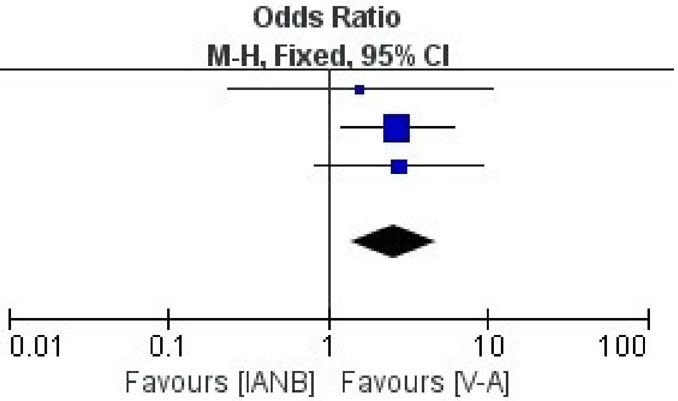

Study or Subgroup	V-A Events	V-A Total	IANB Events	IANB Total	Weight	Risk Ratio M-H, Fixed, 95% CI
Allen, L 1896	18	20	17	20	9.2%	1.06 [0.84, 1.34]
Diandian Li 2009	131	140	118	140	63.8%	1.11 [1.02, 1.21]
Jizhong Lv 2009	56	60	50	60	27.0%	1.12 [0.98, 1.28]
**Total (95% CI)**		**220**		**220**	**100.0%**	**1.11 [1.04, 1.19]**
Total events	205		185			

Heterogeneity: Chi² = 0.17, df = 2 (P = 0.92); I² = 0%
Test for overall effect: Z = 2.97 (P = 0.003)

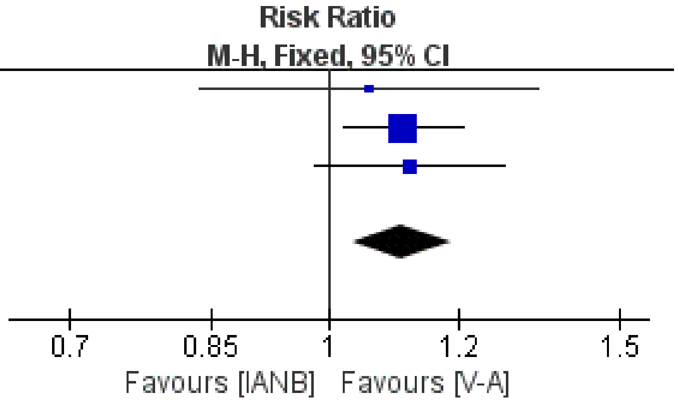

S3

Success rate(G-G vs IANB)

Study or Subgroup	G-G Events	G-G Total	IANB Events	IANB Total	Weight	Odds Ratio M-H, Fixed, 95% CI
Diandian Li 2009	140	140	136	140	21.1%	9.26 [0.49, 173.70]
Fei Wang 2002	29	30	27	30	39.3%	3.22 [0.32, 32.89]
Jieping Yang 2013	31	32	29	32	39.6%	3.21 [0.32, 32.60]
**Total (95% CI)**		**202**		**202**	**100.0%**	**4.49 [1.10, 18.36]**
Total events	200		192			

Heterogeneity: $Chi^2 = 0.39$, df = 2 (P = 0.82); $I^2 = 0\%$
Test for overall effect: Z = 2.09 (P = 0.04)

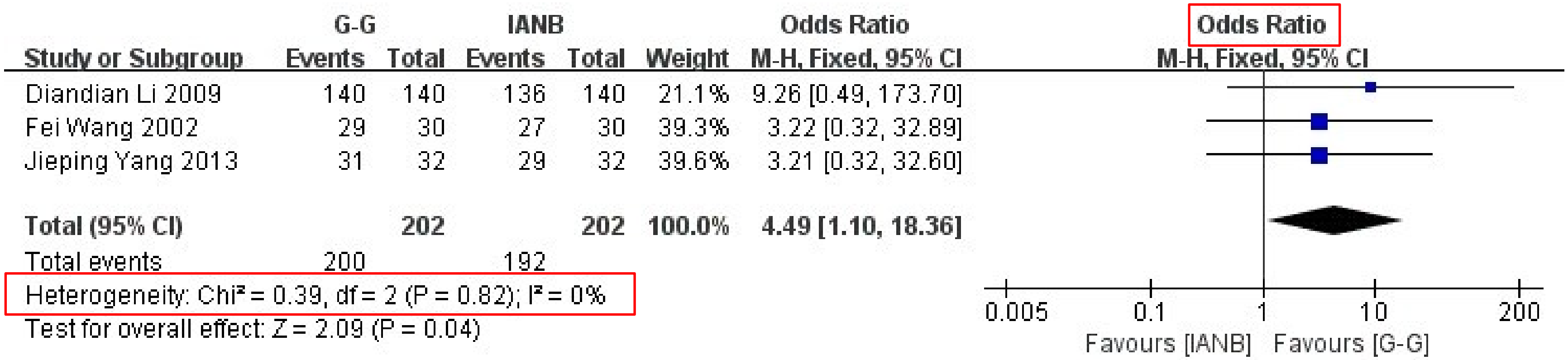

Study or Subgroup	G-G Events	G-G Total	IANB Events	IANB Total	Weight	Risk Ratio M-H, Fixed, 95% CI
Diandian Li 2009	140	140	136	140	70.9%	1.03 [1.00, 1.06]
Jieping Yang 2013	31	32	29	32	15.1%	1.07 [0.94, 1.21]
Fei Wang 2002	29	30	27	30	14.0%	1.07 [0.94, 1.23]
**Total (95% CI)**		**202**		**202**	**100.0%**	**1.04 [1.01, 1.08]**
Total events	200		192			

Heterogeneity: $Chi^2 = 0.89$, df = 2 (P = 0.64); $I^2 = 0\%$
Test for overall effect: Z = 2.26 (P = 0.02)

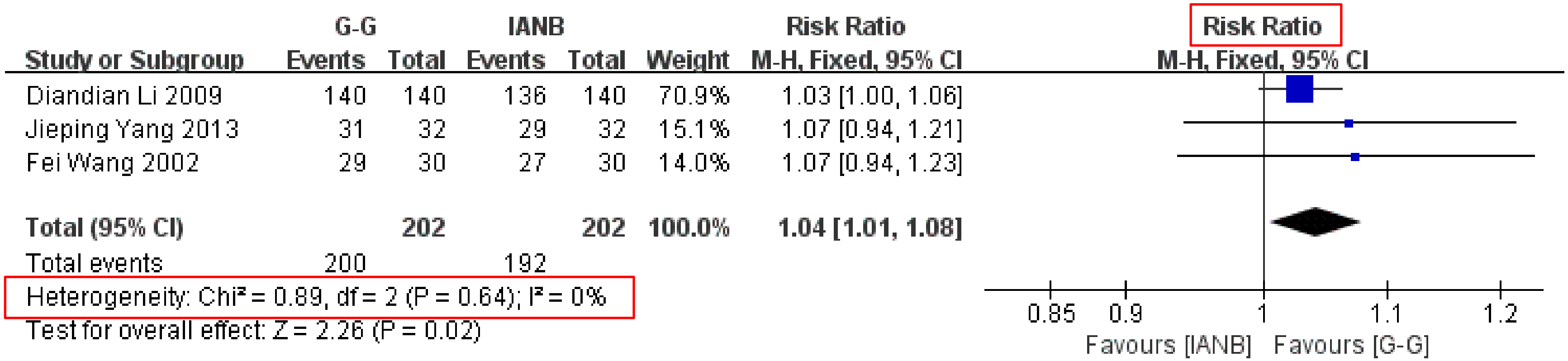

Success rate(V-A vs IANB)

Study or Subgroup	V-A Events	V-A Total	IANB Events	IANB Total	Weight	Odds Ratio M-H, Fixed, 95% CI
Diandian Li 2009	140	140	136	140	6.7%	9.26 [0.49, 173.70]
Jiacai He 2000	38	40	37	40	25.4%	1.54 [0.24, 9.75]
Jizhong Lv 2009	60	60	58	60	6.6%	5.17 [0.24, 110.01]
Martinez-G 2003	23	28	25	28	61.3%	0.55 [0.12, 2.57]
**Total (95% CI)**		**268**		**268**	**100.0%**	**1.69 [0.67, 4.23]**
Total events	261		256			

Heterogeneity: Chi² = 3.85, df = 3 (P = 0.28); I² = 22%
Test for overall effect: Z = 1.12 (P = 0.26)

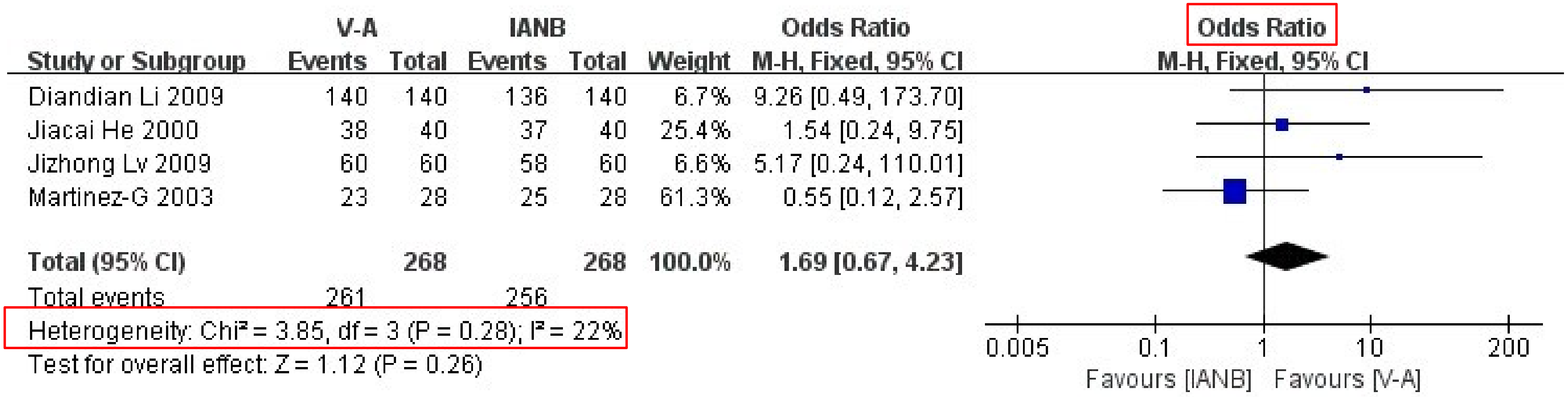

Study or Subgroup	V-A Events	V-A Total	IANB Events	IANB Total	Weight	Risk Ratio M-H, Fixed, 95% CI
Diandian Li 2009	140	140	136	140	53.1%	1.03 [1.00, 1.06]
Jiacai He 2000	38	40	37	40	14.4%	1.03 [0.92, 1.15]
Jizhong Lv 2009	60	60	58	60	22.8%	1.03 [0.98, 1.09]
Martinez-G 2003	23	28	25	28	9.7%	0.92 [0.74, 1.14]
**Total (95% CI)**		**268**		**268**	**100.0%**	**1.02 [0.99, 1.05]**
Total events	261		256			

Heterogeneity: Chi² = 1.49, df = 3 (P = 0.68); I² = 0%
Test for overall effect: Z = 1.13 (P = 0.26)

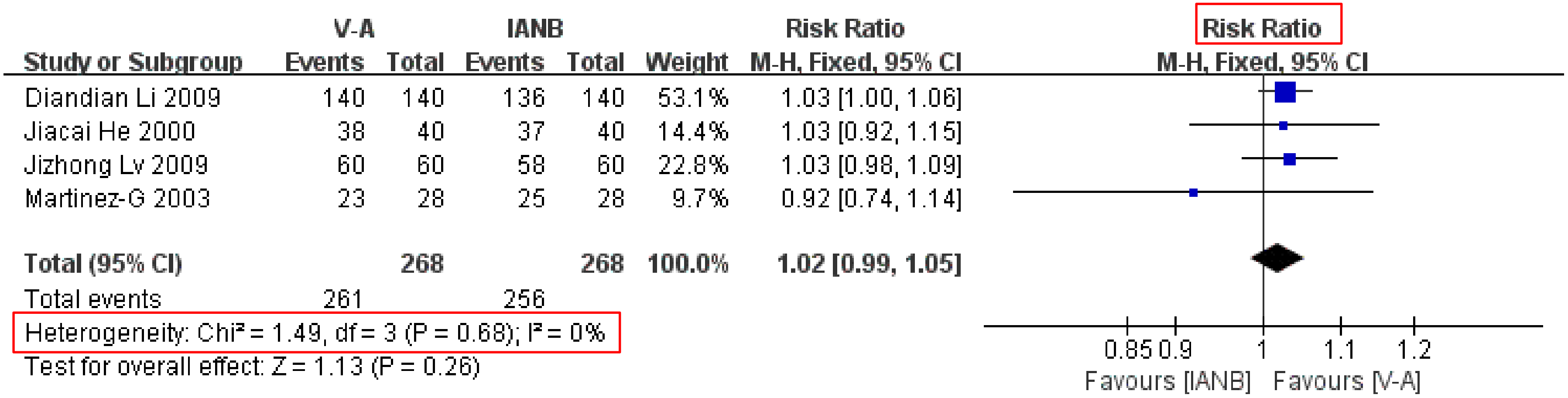

S5

Positive aspiration rate(G-G vs IANB)

Study or Subgroup	G-G Events	G-G Total	IANB Events	IANB Total	Weight	Odds Ratio M-H, Fixed, 95% CI
Diandian Li 2009	0	140	23	140	73.8%	0.02 [0.00, 0.30]
Fei Wang 2002	1	30	3	30	9.1%	0.31 [0.03, 3.17]
Jieping Yang 2013	0	32	5	32	17.1%	0.08 [0.00, 1.45]
**Total (95% CI)**		**202**		**202**	**100.0%**	**0.05 [0.01, 0.23]**
Total events	1		31			

Heterogeneity: Chi² = 2.81, df = 2 (P = 0.25); I² = 29%

Test for overall effect: Z = 3.97 (P < 0.0001)

Odds Ratio
M-H, Fixed, 95% CI

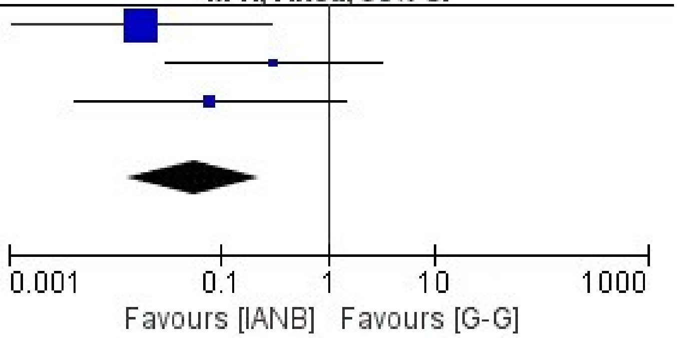

Study or Subgroup	G-G Events	G-G Total	IANB Events	IANB Total	Weight	Risk Ratio M-H, Fixed, 95% CI
Diandian Li 2009	0	140	23	140	73.4%	0.02 [0.00, 0.35]
Fei Wang 2002	1	30	3	30	9.4%	0.33 [0.04, 3.03]
Jieping Yang 2013	0	32	5	32	17.2%	0.09 [0.01, 1.58]
**Total (95% CI)**		**202**		**202**	**100.0%**	**0.06 [0.02, 0.26]**
Total events	1		31			

Heterogeneity: Chi² = 2.85, df = 2 (P = 0.24); I² = 30%

Test for overall effect: Z = 3.83 (P = 0.0001)

Risk Ratio
M-H, Fixed, 95% CI

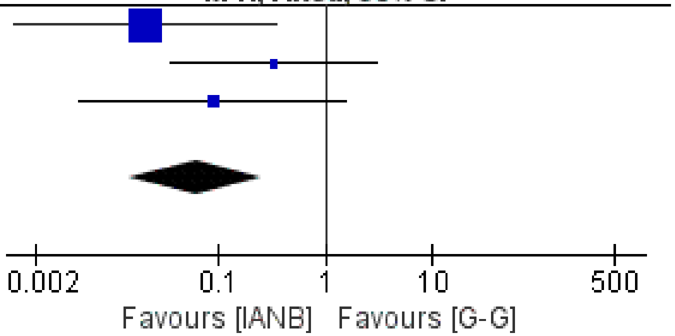

S6

Positive aspiration rate (V-A vs IANB)

Study or Subgroup	V-A Events	V-A Total	IANB Events	IANB Total	Weight	Odds Ratio M-H, Fixed, 95% CI
Diandian Li 2009	0	140	23	140	50.6%	0.02 [0.00, 0.30]
Jiacai He 2000	1	40	4	40	8.4%	0.23 [0.02, 2.16]
Jizhong Lv 2009	0	60	6	60	13.9%	0.07 [0.00, 1.26]
Martinez-G 2003	1	28	13	28	27.1%	0.04 [0.01, 0.36]
**Total (95% CI)**		**268**		**268**	**100.0%**	**0.05 [0.01, 0.16]**
Total events	2		46			

Heterogeneity: Chi² = 2.39, df = 3 (P = 0.50); I² = 0%
Test for overall effect: Z = 4.91 (P < 0.00001)

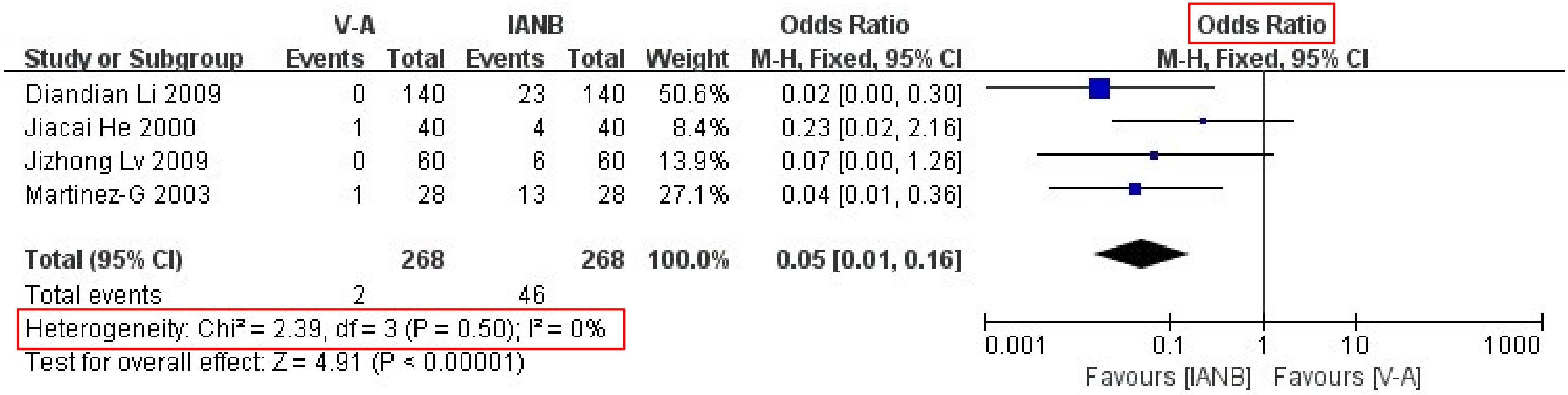

Study or Subgroup	V-A Events	V-A Total	IANB Events	IANB Total	Weight	Risk Ratio M-H, Fixed, 95% CI
Diandian Li 2009	0	140	23	140	50.0%	0.02 [0.00, 0.35]
Jiacai He 2000	1	40	4	40	8.5%	0.25 [0.03, 2.14]
Jizhong Lv 2009	0	60	6	60	13.8%	0.08 [0.00, 1.34]
Martinez-G 2003	1	28	13	28	27.7%	0.08 [0.01, 0.55]
**Total (95% CI)**		**268**		**268**	**100.0%**	**0.06 [0.02, 0.20]**
Total events	2		46			

Heterogeneity: Chi² = 2.20, df = 3 (P = 0.53); I² = 0%
Test for overall effect: Z = 4.69 (P < 0.00001)

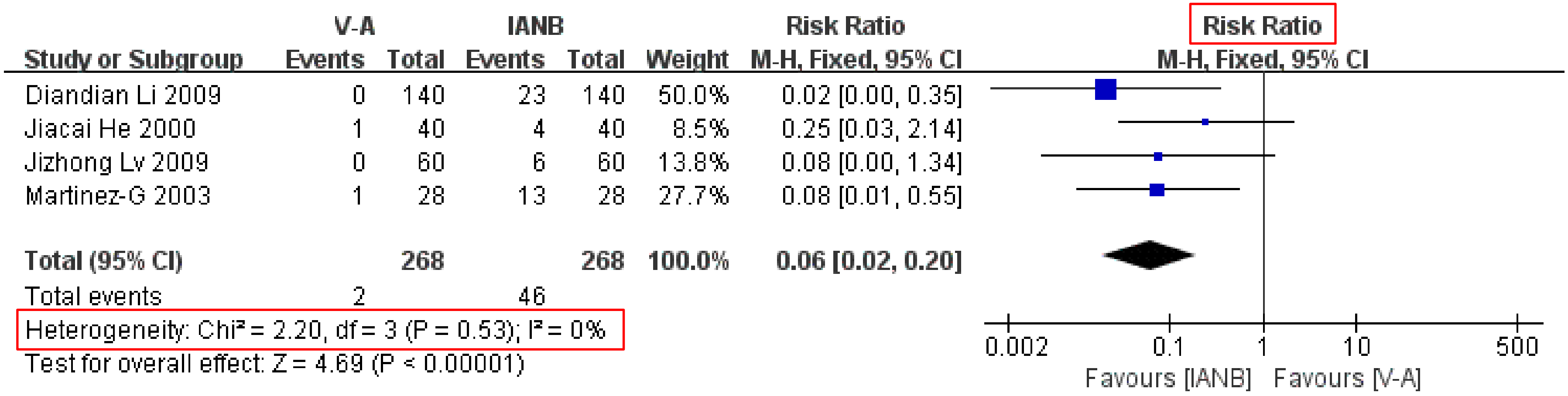

Success rate(G-G vs IANB)

Study or Subgroup	G-G Events	G-G Total	IANB Events	IANB Total	Weight	Risk Ratio M-H, Fixed, 95% CI
Diandian Li 2009	140	140	136	140	70.9%	1.03 [1.00, 1.06]
Jieping Yang 2013	31	32	29	32	15.1%	1.07 [0.94, 1.21]
Fei Wang 2002	29	30	27	30	14.0%	1.07 [0.94, 1.23]
**Total (95% CI)**		**202**		**202**	**100.0%**	**1.04 [1.01, 1.08]**
Total events	200		192			

Heterogeneity: Chi² = 0.89, df = 2 (P = 0.64); I² = 0%
Test for overall effect: Z = 2.26 (P = 0.02)

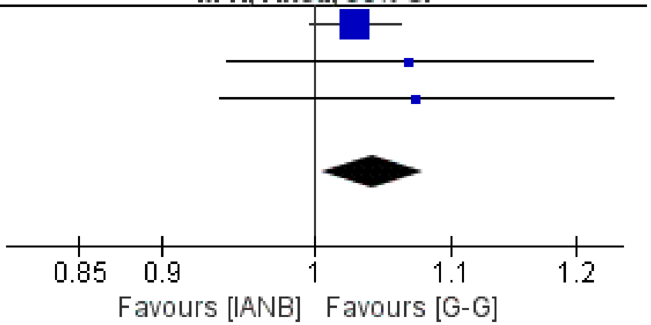

Study or Subgroup	G-G Events	G-G Total	IANB Events	IANB Total	Weight	Risk Ratio M-H, Fixed, 95% CI
Diandian Li 2009	140	140	136	140	83.5%	1.03 [1.00, 1.06]
Fei Wang 2002	29	30	27	30	16.5%	1.07 [0.94, 1.23]
**Total (95% CI)**		**170**		**170**	**100.0%**	**1.04 [1.00, 1.07]**
Total events	169		163			

Heterogeneity: Chi² = 0.46, df = 1 (P = 0.50); I² = 0%
Test for overall effect: Z = 2.03 (P = 0.04)

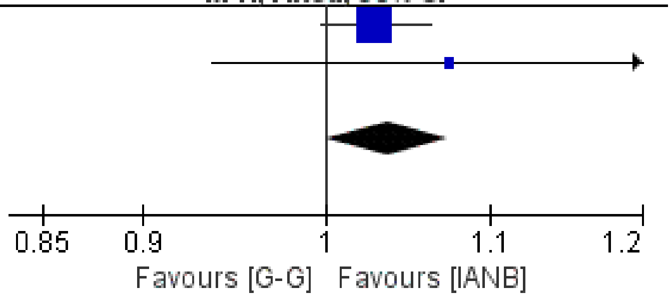

S8

Success rate(G-G vs IANB)

	V-A		IANB			Risk Ratio
Study or Subgroup	Events	Total	Events	Total	Weight	M-H, Fixed, 95% CI
Diandian Li 2009	140	140	136	140	53.1%	1.03 [1.00, 1.06]
Jiacai He 2000	38	40	37	40	14.4%	1.03 [0.92, 1.15]
Jizhong Lv 2009	60	60	58	60	22.8%	1.03 [0.98, 1.09]
Martinez-G 2003	23	28	25	28	9.7%	0.92 [0.74, 1.14]
**Total (95% CI)**		**268**		**268**	**100.0%**	**1.02 [0.99, 1.05]**
Total events	261		256			

Heterogeneity: Chi² = 1.49, df = 3 (P = 0.68); I² = 0%
Test for overall effect: Z = 1.13 (P = 0.26)

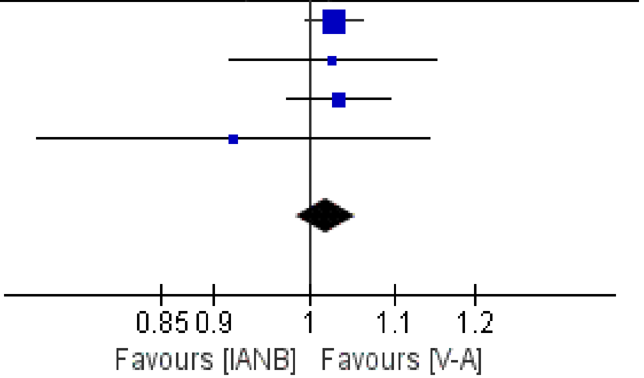

	V-A		IANB			Risk Ratio
Study or Subgroup	Events	Total	Events	Total	Weight	M-H, Fixed, 95% CI
Diandian Li 2009	140	140	136	140	62.0%	1.03 [1.00, 1.06]
Jizhong Lv 2009	60	60	58	60	26.6%	1.03 [0.98, 1.09]
Martinez-G 2003	23	28	25	28	11.4%	0.92 [0.74, 1.14]
**Total (95% CI)**		**228**		**228**	**100.0%**	**1.02 [0.98, 1.05]**
Total events	223		219			

Heterogeneity: Chi² = 1.60, df = 2 (P = 0.45); I² = 0%
Test for overall effect: Z = 1.04 (P = 0.30)

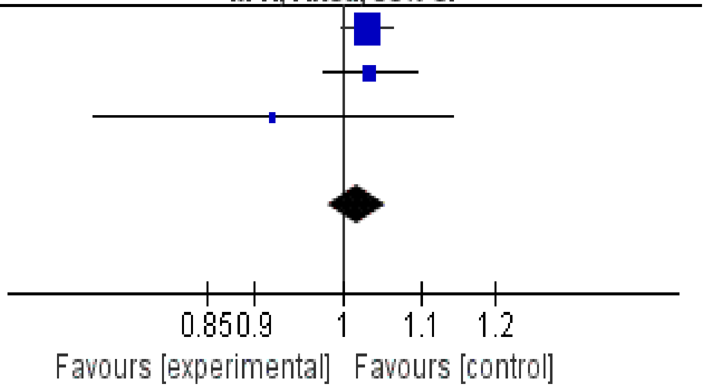

Onset time(G-G vs IANB)

	G-G		IANB			Risk Ratio
Study or Subgroup	Events	Total	Events	Total	Weight	M-H, Fixed, 95% CI
Diandian Li 2009	69	140	118	140	84.3%	0.58 [0.49, 0.70]
Fei Wang 2002	10	30	22	30	15.7%	0.45 [0.26, 0.79]
**Total (95% CI)**		**170**		**170**	**100.0%**	**0.56 [0.47, 0.67]**
Total events	79		140			

Heterogeneity: Chi² = 0.74, df = 1 (P = 0.39); I² = 0%

Test for overall effect: Z = 6.41 (P < 0.00001)

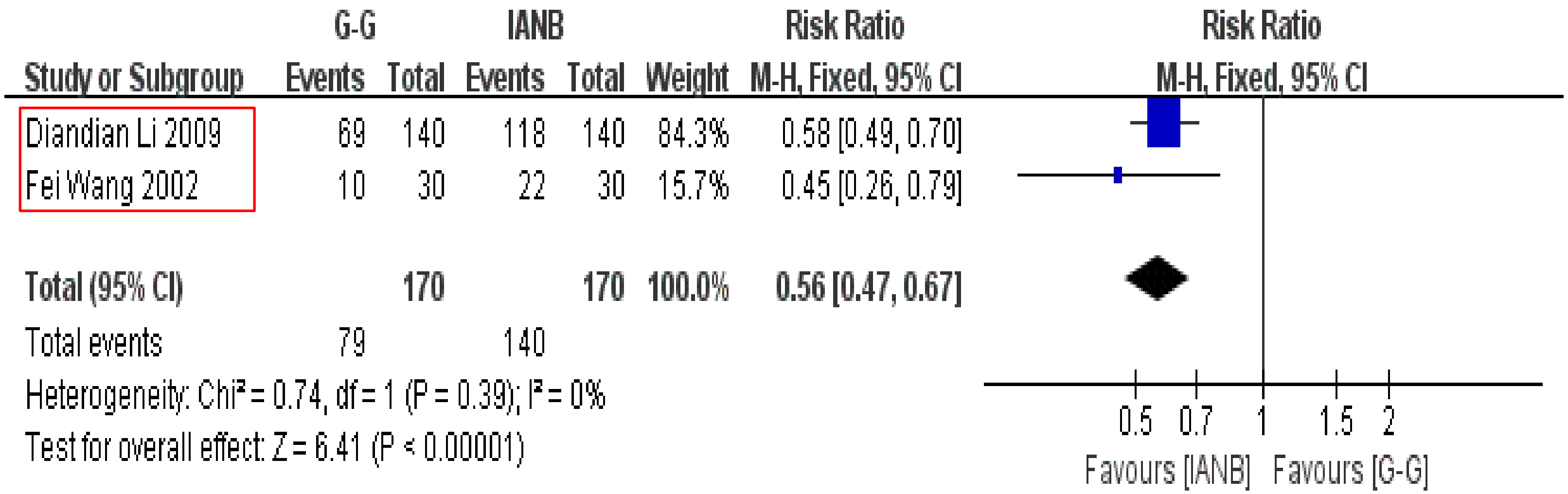

S10

Onset time(V-A vs IANB)

Study or Subgroup	V-A Events	V-A Total	IANB Events	IANB Total	Weight	Risk Ratio M-H, Fixed, 95% CI
Allen, L 1896	18	20	17	20	9.2%	1.06 [0.84, 1.34]
Diandian Li 2009	131	140	118	140	63.8%	1.11 [1.02, 1.21]
Jizhong Lv 2009	56	60	50	60	27.0%	1.12 [0.98, 1.28]
**Total (95% CI)**		**220**		**220**	**100.0%**	**1.11 [1.04, 1.19]**
Total events	205		185			
Heterogeneity: Chi² = 0.17, df = 2 (P = 0.92); I² = 0%						
Test for overall effect: Z = 2.97 (P = 0.003)						

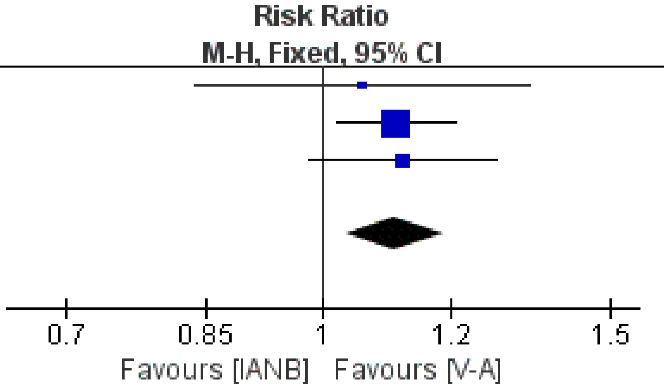

Study or Subgroup	V-A Events	V-A Total	IANB Events	IANB Total	Weight	Risk Ratio M-H, Fixed, 95% CI
Diandian Li 2009	131	140	118	140	70.2%	1.11 [1.02, 1.21]
Jizhong Lv 2009	56	60	50	60	29.8%	1.12 [0.98, 1.28]
**Total (95% CI)**		**200**		**200**	**100.0%**	**1.11 [1.04, 1.19]**
Total events	187		168			
Heterogeneity: Chi² = 0.01, df = 1 (P = 0.91); I² = 0%						
Test for overall effect: Z = 2.97 (P = 0.003)						

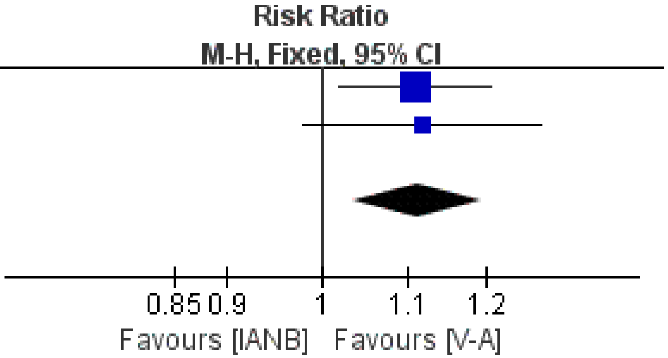

Positive aspiration rate(G-G vs IANB)

Study or Subgroup	G-G Events	G-G Total	IANB Events	IANB Total	Weight	Risk Ratio M-H, Fixed, 95% CI
Diandian Li 2009	0	140	23	140	73.4%	0.02 [0.00, 0.35]
Fei Wang 2002	1	30	3	30	9.4%	0.33 [0.04, 3.03]
Jieping Yang 2013	0	32	5	32	17.2%	0.09 [0.01, 1.58]
**Total (95% CI)**		**202**		**202**	**100.0%**	**0.06 [0.02, 0.26]**
Total events	1		31			

Heterogeneity: Chi^2 = 2.85, df = 2 (P = 0.24); I^2 = 30%
Test for overall effect: Z = 3.83 (P = 0.0001)

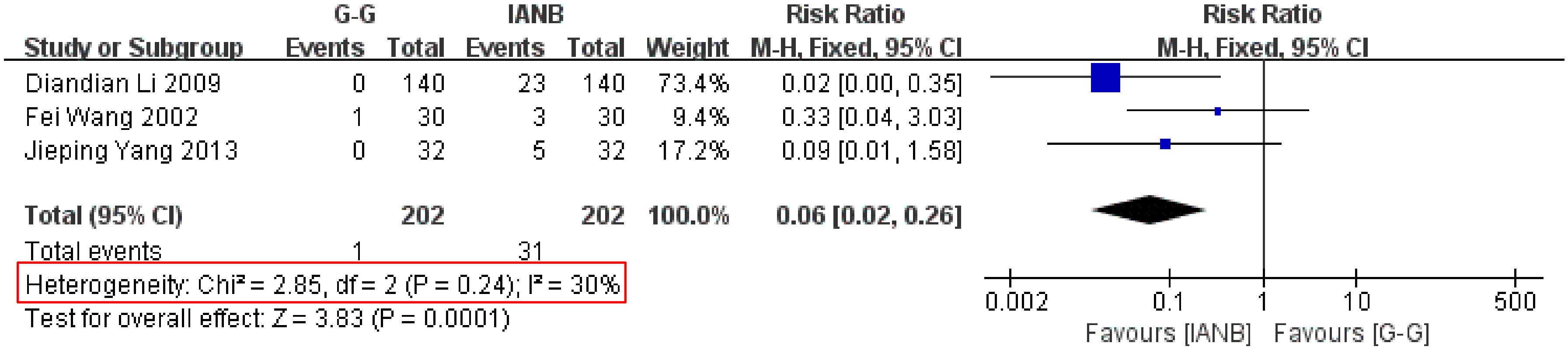

Study or Subgroup	G-G Events	G-G Total	IANB Events	IANB Total	Weight	Risk Ratio M-H, Random, 95% CI
Diandian Li 2009	0	140	23	140	46.1%	0.02 [0.00, 0.35]
Fei Wang 2002	1	30	3	30	53.9%	0.33 [0.04, 3.03]
**Total (95% CI)**		**170**		**170**	**100.0%**	**0.09 [0.00, 1.98]**
Total events	1		26			

Heterogeneity: Tau^2 = 3.22; Chi^2 = 2.95, df = 1 (P = 0.09); I^2 = 66%
Test for overall effect: Z = 1.52 (P = 0.13)

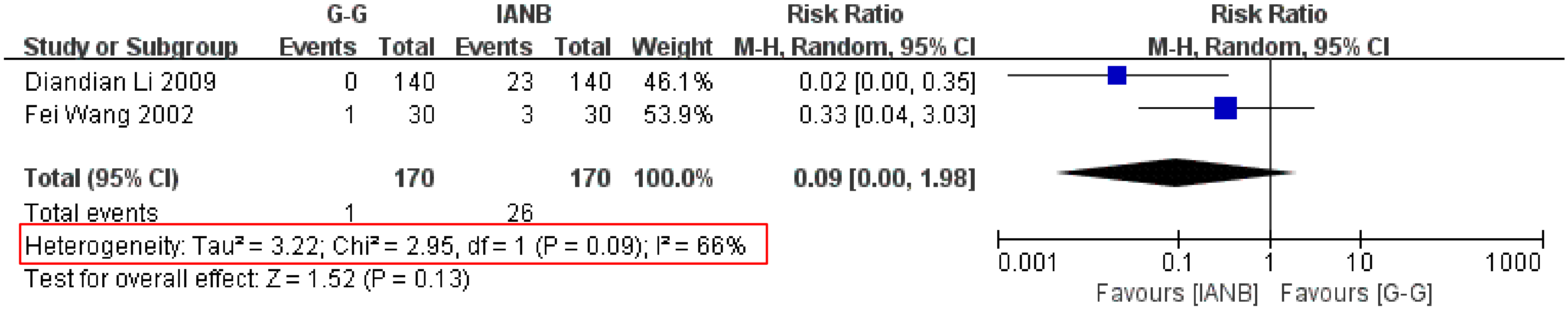

Positive aspiration(V-A vs IANB) S12

Study or Subgroup	V-A Events	V-A Total	IANB Events	IANB Total	Weight	Risk Ratio M-H, Fixed, 95% CI
Diandian Li 2009	0	140	23	140	50.0%	0.02 [0.00, 0.35]
Jiacai He 2000	1	40	4	40	8.5%	0.25 [0.03, 2.14]
Jizhong Lv 2009	0	60	6	60	13.8%	0.08 [0.00, 1.34]
Martinez-G 2003	1	28	13	28	27.7%	0.08 [0.01, 0.55]
**Total (95% CI)**		**268**		**268**	**100.0%**	**0.06 [0.02, 0.20]**
Total events	2		46			

Heterogeneity: Chi^2 = 2.20, df = 3 (P = 0.53); I^2 = 0%
Test for overall effect: Z = 4.69 (P < 0.00001)

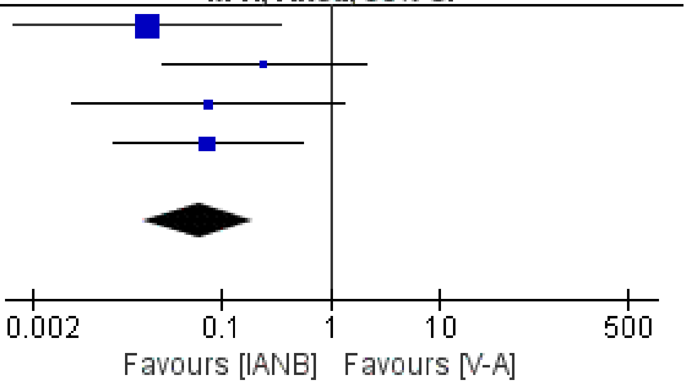

Study or Subgroup	V-A Events	V-A Total	IANB Events	IANB Total	Weight	Risk Ratio M-H, Fixed, 95% CI
Diandian Li 2009	0	140	23	140	54.7%	0.02 [0.00, 0.35]
Jizhong Lv 2009	0	60	6	60	15.1%	0.08 [0.00, 1.34]
Martinez-G 2003	1	28	13	28	30.2%	0.08 [0.01, 0.55]
**Total (95% CI)**		**228**		**228**	**100.0%**	**0.05 [0.01, 0.19]**
Total events	1		42			

Heterogeneity: Chi^2 = 0.67, df = 2 (P = 0.71); I^2 = 0%
Test for overall effect: Z = 4.30 (P < 0.0001)

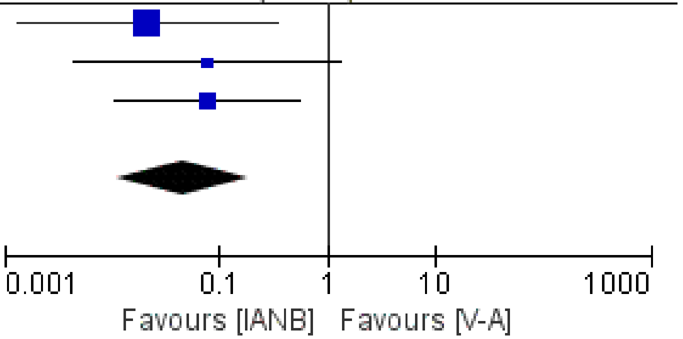

Flow Diagram

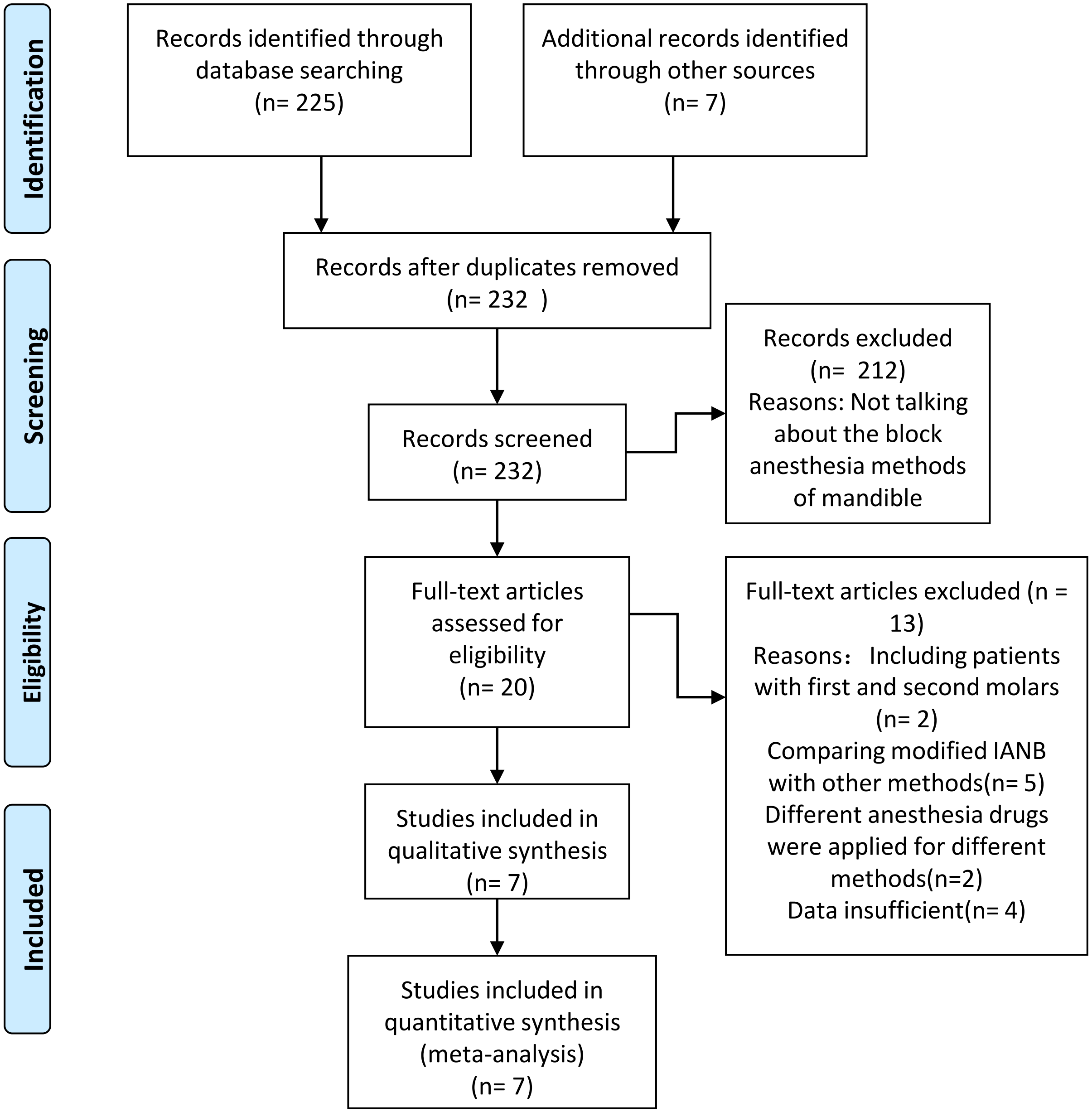

Supplement: Supplemental Data [file srep40987-s1.pdf]
